# Near-Infrared Light-Responsive Nitric Oxide Delivery Platform for Enhanced Radioimmunotherapy

**DOI:** 10.1007/s40820-020-00431-3

**Published:** 2020-04-24

**Authors:** Xuanfang Zhou, Zhouqi Meng, Jialin She, Yaojia Zhang, Xuan Yi, Hailin Zhou, Jing Zhong, Ziliang Dong, Xiao Han, Muchao Chen, Qin Fan, Kai Yang, Chao Wang

**Affiliations:** 1 Institute of Functional Nano and Soft Materials (FUNSOM), Jiangsu Key Laboratory for Carbon-Based Functional Materials and Devices, Soochow University, Suzhou, 215123 Jiangsu People’s Republic of China; 2 State Key Laboratory of Radiation Medicine and Protection, School of Radiation Medicine and Protection and School for Radiological and Interdisciplinary Sciences (RAD-X), Collaborative Innovation Center of Radiation Medicine of Jiangsu Higher Education Institutions, Soochow University, Suzhou, 215123 Jiangsu People’s Republic of China

**Keywords:** NO delivery, Radio sensitivity, Multifunctional agent, Radioimmunotherapy, Drug delivery

## Abstract

Radiotherapy (RT) is a widely used way for cancer treatment. However, the efficiency of RT may come with various challenges such as low specificity, limitation by resistance, high dose and so on. Nitric oxide (NO) is known a very effective radiosensitizer of hypoxic tumor. However, NO cannot circulate in body with high concentration. Herein, an NIR light-responsive NO delivery system is developed for controlled and precisely release of NO to hypoxic tumors during radiotherapy. *Tert*-Butyl nitrite, which is an efficient NO source, is coupled to Ag_2_S quantum dots (QDs). NO could be generated and released from the Ag_2_S QDs effectively under the NIR irradiation due to the thermal effect. In addition, Ag is also a type of heavy metal that can benefit the RT therapy. We demonstrate that Ag_2_S NO delivery platforms remarkably maximize radiotherapy effects to inhibit tumor growth in CT26 tumor model. Furthermore, immunosuppressive tumor microenvironment is improved by our NO delivery system, significantly enhancing the anti-PD-L1 immune checkpoint blockade therapy. 100% survival rate is achieved by the radio-immune combined therapy strategy based on the Ag_2_S NO delivery platforms. Our results suggest the promise of Ag_2_S NO delivery platforms for multifunctional cancer radioimmunotherapy.

**Electronic supplementary material:**

The online version of this article (10.1007/s40820-020-00431-3) contains supplementary material, which is available to authorized users.

## Highlights

- Ag_2_S NO delivery platforms maximize radiotherapy effects remarkably to inhibit the tumor growth.
- Immunosuppressive tumor microenvironment was improved by Ag_2_S NO delivery system, significantly enhancing the anti-PD-L1 immune checkpoint blockade therapy.

**Electronic supplementary material:**

The online version of this article (10.1007/s40820-020-00431-3) contains supplementary material, which is available to authorized users.

## Graphical abstract

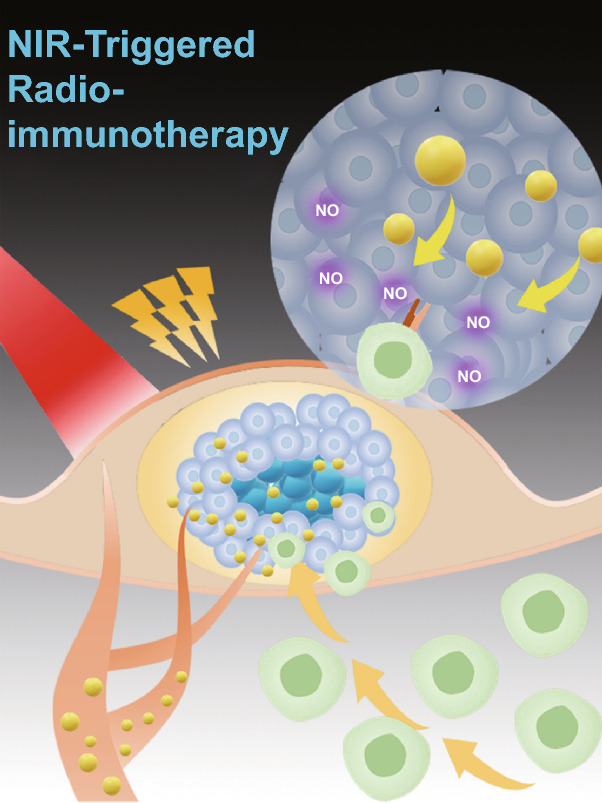

## Introduction

Radiotherapy (RT), which uses radiation to kill cancer cells by damaging DNA, is widely used in clinical applications [1–4]. As one type of RT, external beam radiotherapy is a localized therapy that applies high-energy beams to target directly at the tumor. However, the efficacy of RT can be influenced by the tumor hypoxic microenvironment, resulting in the resistance of tumor to RT [4, 5]. Meanwhile, various severe side effects associated with RT are still one of major challenges in clinical practice. How to make cancer cells more sensitive to RT, whilst avoiding or minimizing damage to surrounding healthy tissue is urgent to be addressed [6, 7].

In addition to many heavy metals (e.g., gold, platinum, silver) that could enlargement radiation effects, nitric oxide (NO) is known a very effective radiosensitizer of hypoxic tumor as well [8–13]. It has previously been shown that DNA damage induced by NO during radiation is remarkably enhanced [2, 9, 14–18]. Recent studies also indicate that NO can regulate the functional of many immune cells including macrophages, T lymphocytes and antigen-presenting cells, making them more active against the infection and cancer [19–21]. However, NO cannot circulate in body with high concentration (half-life is less than 5 s and diffusion radius is less than 40 µm) and it can participate in many pathways to disturb internal environment due to their high bioactivity [13, 17, 22–27]. A potential strategy to develop a system for controlled and precisely release of NO to hypoxic tumors during radiotherapy holds great promise to improve the RT [7, 16, 28–30].

**Fig. 1:**
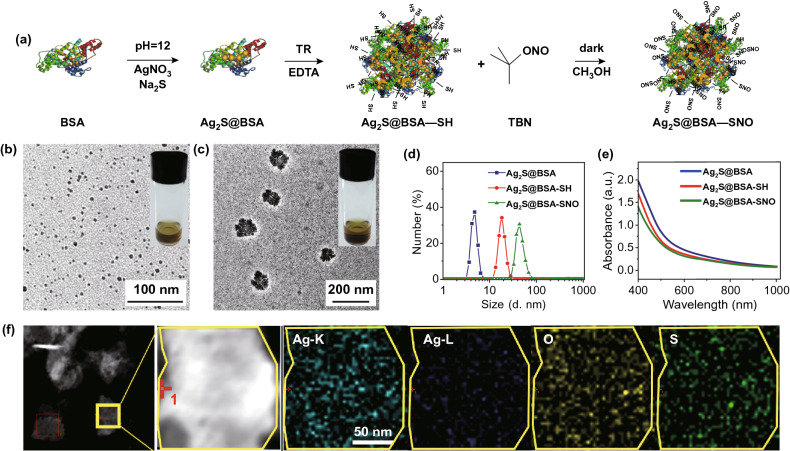
Schematic illustration and characterization of Ag_2_S@BSA-SNO nanoparticles. **a** A schematics illustration showing Ag_2_S growth in BSA nanocage and NO donor function group formation. **b, c** TEM images of Ag_2_S@BSA and Ag_2_S@BSA-SNO. Inset: picture of Ag_2_S@BSA and Ag_2_S@BSA-SNO solution (20 mg mL^−1^). **d** Dynamic light scattering (DLS) analysis of Ag_2_S@BSA, Ag_2_S@BSA-SH and Ag_2_S@BSA-SNO. **e** UV–Vis–NIR spectra of Ag_2_S@BSA, Ag_2_S@BSA-SH and Ag_2_S@BSA-SNO solution. **f** STEM image of Ag_2_S@BSA-SNO nanoparticles to show elements’ locations

Numerous NO donors can be used to deliver and release NO by various triggers, such as pH, heat and light [31–42]. Semiconductor metal sulfide materials such as Ag_2_S quantum dots (QDs) have been widely used in optical imaging, photoacoustic imaging, sensing and photothermal therapy [43]. Ag is also a type of heavy metal that can benefit the RT therapy. Here, we described an NIR-induced, thermal-triggered NO release system based on the Ag_2_S QDs for the improvement in RT. In our system, proteins (such as BSA or OVA) were used to modify the Ag_2_S QDs; then, the NO donor *tert*-butyl nitride (TBN) could be further coupled to the proteins to form Ag_2_S@BSA-SNO nanoparticles (Fig. 1a).We demonstrated that NO could be generated and released from the Ag_2_S QDs effectively under the NIR irradiation in vitro [43–47]. In the mice tumor model, Ag_2_S QDs could accumulate at the tumor site by EPR effect. We demonstrated that NIR irradiation made tumor more sensitive to the following RT. Synergetic effects were achieved, which could eliminate most established CT26 tumor in nude mice. Moreover, we further demonstrated that this strategy could induce specific antitumor immune response in B6 mice bearing B16F10 tumor, significantly increasing anti-PD-L1 therapy response, making our Ag_2_S QDs platform promise in cancer radioimmunotherapy.

## Materials and Methods

### Materials

Albumin from bovine serum (BSA) was bought from Beijing J&K Scientific. *Tert*-butyl nitride (TBN) was provided by Alfa Aesar. Silver nitrate (AgNO_3_), sodium sulfide (Na_2_S), EDTA (ethylenediaminetetraacetic acid) and sodium hydroxide (NaOH), methyl alcohol and dichloromethane were bought from China National Pharmaceutical Industry Corporation Ltd. PBS buffer was got from Solarbio. Traut’s reagent was provided by BioVision. Nitric oxide assay kit was bought from Beyotime. Ultrapure water was obtained from a Milli-Q system. Antibody was bought from BioLegend and Invitrogen.

### Preparation and Characteristic of Ag_2_S@BSA-SNO Nanoparticles

#### Synthesis of Ag_2_S@BSA

According to the previous work, Ag_2_S@BSA was produced by mineralization of bovine serum albumin. In brief, 250 mg BSA powder was dispersed in 8 mL ultrapure water, and then 2 mL 0.2 mM AgNO_3_ solution was dropped in BSA solutions. The system became turbid. NaOH solutions were used to regulate the whole system’s pH value around 12 to make a suitable condition for crystal growth. Finally, there was 18.2 mg Na_2_S which provides anion for Ag_2_S crystal added. With reaction going on, color of solution changed from yellow to sepia. After 4-h stirring under 55 °C, Ag_2_S@BSA solution was obtained.

#### Synthesis of Ag_2_S@BSA-SH

Ag_2_S@BSA solution containing 10 mg BSA was added to 5 mL 5 mM EDTA solution (dissolved by PBS buffer) and then added 1 mg Traut’s reagent for stirring 2 h under 4 °C to modify surface function group of BSA nanoparticles. The production got from this procedure would be dialysis in DI water to remove extra saline ions.

#### Synthesis of Ag_2_S@BSA-SNO

The concentration of Ag_2_S@BSA-SH solution was concentrated through ultrafiltration with a 100 kD Millipore to decrease the volume of water. Methyl and dichloromethane (${\text{V}}_{{{\text{MeOH}}}} /{\text{V}}_{{{\text{CH}}_{2} {\text{Cl}}_{2} }} = 5$) were mixed to dissolve TBN, which was the nitric oxide donor. Condensed Ag_2_S@BSA-SH solution with 10 mg BSA was added in 5 mL methyl when stirring. 1 mL TBN solution was mixed with methyl/dichloromethane solution and then added to Ag_2_S@BSA-SH solution. The following reaction was kept in dark environment and under 4 °C. Finally, organic solvent was removed by rotary evaporation and then Ag_2_S@BSA-SNO was re-dissolved by PBS and then stored in 4 °C. During the process, free Ag_2_S was removed during the dialysis process, while the extra TBN was removed with organic solvent during the rotary evaporation [16].

### Characterization

Dynamic light scattering (DLS) was measured by Malvern, NANO ZS90, while TEM images were got from Tecnai G2 F20 field emission transmission electron microscopy. The UV–Vis spectra of different concentrations of Ag_2_S@BSA-SNO solutions were detected by ultraviolet and visible spectrophotometer, and the concentration was determined by silver anion. The photoacoustic signals of materials were measured by the PA mode of PA imaging system (FUJIFILM VisualSonics Inc.).

### NO Generation Tests

The production of NO was tested by nitric oxide assay kit. In short, Ag_2_S@BSA-SNO samples were incubated under 37 °C or laser-irradiated which caused different temperatures. The released NO would convert into nitrite; after that, the Griess agent reacted with nitrite to form diazo compound. Finally, the signal of diazo compound was detected by microplate reader at 540 nm. There were at least three parallel samples at each condition.

### NIR Photothermal Heating

Ag_2_S@BSA-SNO nanoparticles with different concentrations in PBS buffer were irradiated by 808 nm semiconductor laser device for 5 min (1.0 W cm^−2^). And the real-time temperature change was monitored by a thermal imaging temperature monitoring system FLIR-A300 (FILR Systems Inc.).

### Cell Experiment

#### Cell Culture

The CT26 murine colonic cancer cells were cultured in RPMI-1640 culture medium in a humidified incubator at 37 °C under 5% CO_2_. The 1640 culture medium was added with 10% fetal bovine serum (FBS) and 1% penicillin/ streptomycin, while the B16F10 murine melanoma cells were cultured in DMEM/high-glucose culture medium that contained 10% FBS and 1% penicillin/streptomycin in one incubator.

#### Cytotoxicity Experiment

The viability of CT26 cells exposed to Ag_2_S@BSA-SNO and photothermal effect was assessed by methyl thiazolyl tetrazolium (MTT) assay. 10^4^ CT26 cells were pre-cultured in 96-well plate for 24 h before adding Ag_2_S@BSA-SNO solution. After that, RPMI-1640 was removed and new culture was added with different concentrations of Ag_2_S@BSA-SNO solution and co-cultured for another 24 h. Before adding MTT solution, all solutions were removed and 96-well plate was washed twice with PBS buffer. After 4-h incubation, MTT solution was removed carefully, 100 µL DMSO was added per well, and the absorption was tested at 570 nm by a microplate reader after 10-min vibration. Each condition was performed with six replicates.

The hemolytic assay was performed using fresh mice red blood cells. PBS was used to re-suspend RBCs. Ag_2_S@BSA-SNO NPs were then added to RBC solution, and the same volume of water was set as positive control. The supernatant was obtained by centrifuging samples 1 h later under 37 °C. UV absorbance of supernatant at 545 nm was measured and recorded. The hemolysis ratio was calculated by division of the difference between experimental group/positive control group and negative control group.

#### PI/AM Double Staining

10^6^ cells per 35-mm dish were incubated for 24 h before any treatment. And then, added Ag_2_S@BSA solution for a final concentration is 100 µg mL^−1^. About 4-h co-culturing, the cell was lighted by 808 nm laser (1.5 W cm^−2^, 42 °C). The cells after different processing time were washed with PBS for several times to remove all materials. And then the cells were co-stained with calcein AM and propidium iodide (PI) for 20 min to identify if they were living or dead. Then, the cells were imaged by confocal fluorescence microscope (Zeiss).

#### Cell Clonogenic Experiment

Different mounts of CT26 cells (125, 1000 cells per well corresponding to 0 Gy and 6 Gy) were pre-seeded into 6-well plates. After 24 h cultured as usual at normal environment, Ag_2_S@BSA or Ag_2_S@BSA-SNO was added to incubate cells for another 4 h. After that, plates were exposed to irradiation of laser device (1.5 W cm^−2^, 42 °C, 5 min) or X-ray (6 Gy) or both of them. Next, fresh RPMI-1640 medium (1% penicillin/streptomycin, 10% fetal calf serum) was used to culture cells for additional 7 days. Finally, cells were rinsed with PBS for three times and fixed by methyl for around 15 min to be colored with crystal violet about 30 min. And then, cell colonies were counted and calculated for surviving fraction = (survival colonies)/(seeded cells) × %. The average survival was calculated by three replicates.

#### Cell γ-H2AX Immunofluorescence Staining

In the experiment of CT26 cells γ-H2AX immunofluorescence staining, 10^4^/mL CT26 cells were pre-seeded in a 24-well plate. And after 18 h being cultured, cells were added with different kinds of solutions (Ag_2_S@BSA, Ag_2_S@BSA-SNO or same volume of PBS). After co-cultured lasted for 6 h, cells received laser irradiation or X-ray irradiation or both of them. The power of 808 nm laser was 1.5 W cm^−2^ (5 min), while the X-ray irradiation dose was 6 Gy. After 60 min, the cells were washed with PBS and fixed by 4% formaldehyde for 15 min. 1% bovine serum albumin was used as blocking solution for treating cells about 1 h. After that, the blocking solution was removed and anti-phospho-histone γ-H2AX mouse monoclonal antibody (dilution was 1:1000) was used to stain cells at 4 °C overnight. After extra antibody was washed with PBS, cells were treated by Cy3-conjugted secondary antibody at 37 °C for 1 h. Cells were imaged by a confocal fluorescence scanning microscopy (Zeiss). The area % of DNA damage in every sample was analyzed by ImageJ software.

### Animal Experiment

C57BL/6 and BALB/c Nude mice (6–10 weeks) were purchased from Changzhou Cavens Experimental Animal Co. Ltd. Mice were treated under protocols approved by the Institutional Animal Care and Use Committee of Soochow University.

#### PA Imaging

CT26 tumor-bearing nude mice were i.v. injected with 200 µL Ag_2_S@BSA-SNO nanoparticles (20 mg mL^−1^) solution or PBS. And then, the tumor region was imaged with an PA imaging system under 730 nm excitation wavelength at different time intervals after injection (VisualSonics Vevo 2100 LAZER).

#### IR Thermal Imaging In Vivo

After 24-h injection of 200 µL Ag_2_S@BSA-SNO, Ag_2_S@BSA or PBS solutions, CT26 tumor-bearing mice were irradiated by 808 nm laser device with 1.0 W cm^−2^ power and lasted for about 10 min to do temperature change recording. IR images were monitored timely by the IR thermal camera.

#### Biodistribution and Blood Circulation

CT26-bearing nude mice were intravenous injected with 200 µL 10 mg mL^−1^ Ag_2_S@BSA-SNO solution. Blood samples were obtained through vena ophthalmic at specific time points. Organs were obtained after one day post-injection. And then, aqua regia was used to dissolve tissues and blood samples. The clear solution was filtered by 0.22-µm filter membrane. Whereafter, the concentration of Ag^+^ was detected by inductively coupled plasma source mass spectrometer (Aurora M90). And the values of ID% were calculated by concentration of Ag^+^ to the mass of tissue or blood.

#### Immunohistochemistry

Tumor region of mice was irradiated by 808 nm laser (1.0 W cm^−2^) about 10 min; then, the mice were injected with pimonidazole hydrochloride (60 mg kg^−1^, Hypoxiaprobe-1 Plus Kit, Hypoxyprobe Inc.) immediately. The Hypoxiaprobe-1 was injected by intraperitoneal injection. And after 30-min post-injection, tumor was dissected and fixed in O.C.T. glue under − 80 °C. For further immunofluorescence staining, tumors were cut into 10-µm sections through frozen cutting. And then the sections were fixed by acetone and blocked by 1% BSA solution. Hypoxyprobe anti-pimonidazole primary antibody and 488-conjugated goat anti-mouse secondary antibody was stained in the light of kit’s introductions. Anti-CD31 mouse monoclonal primary antibody and rhodamine-conjugated secondary antibody were used to stain blood vessels. Nuclei were dyed by DAPI. Extra antibody was washed with PBS. Finally, sections were scanned by confocal fluorescence microscope (Zeiss) to get the image.

#### Tumor Treatment In Vivo

When tumor size achieved about 125 mm^3^, nude mice bearing CT26 tumor were divided into eight groups (five mice per group): (1) untreated; (2) X-ray irradiation; (3) Ag_2_S@BSA-SNO; (4) Ag_2_S@BSA + laser; (5) Ag_2_S@BSA-SNO + X-ray; (6) Ag_2_S@BSA-SNO + laser; (7) Ag_2_S@BSA + laser + X-ray; and (8) Ag_2_S@BSA-SNO + laser + X-ray. Each mouse was injected with 200 µL solutions (Ag_2_S@BSA or Ag_2_S@BSA-SNO, respectively, 20 mg mL^−1^) through caudal vein. After 4 h, mice in groups 4, 6, 7 and 8 were exposed to 808 nm laser (1 W cm^−2^, 42 °C, 10 min), while mice in groups 2, 5 and 8 which received radiotherapy were exposed to X-ray (6 Gy). Particularly, groups 7 and 8 that received both of the treatments were irradiated by 808 nm laser first. The body weight and tumors’ size were measured at day 0 before treatment. The body weight and tumor size were recorded every two days until the tumor size of one mouse in a group reached over 1000 mm^3^.

#### Immunotherapy and Immunological Evaluation of Ag_2_S@OVA-SNO-Treated B16F10 Tumor In Vivo

For in vivo immunotherapy, B16F10 tumor model was built on B6 mice. When tumor size was around 100 mm^3^, mice were divided into four groups (eight mice per groups): (1) untreated; (2) α-PD-L1; (3) Ag_2_S@OVA-SNO + laser + X-ray; and (4) Ag_2_S@OVA-SNO + laser + X-ray + α-PD-L1. Mice in group 2 were injected with α-PD-L1 (1 mg kg^−1^, three injections during 7 days), while group 4 needed injection α-PD-L1 as well. Mice in group 3 and group 4 were injected with 25 µL Ag_2_S@OVA-SNOsolution (5 mg mL^−1^), and then tumor was irradiated by 808 nm laser (1 W cm^−2^, 42 °C, 10 min). After laser irradiation, mice were exposed to X-ray (6 Gy). Tumor size and body weight were monitored every two days.

At day 4 after treatment, three mice from every group were mercy euthanatized and blood samples and tumor tissue were collected for immunological evaluation. A part of tumor tissue was fixed by O.C.T. glue to make frozen section for immunological staining. And the last part was homogenized into single-cell suspensions according to the well-established protocol. Those cells were stained with anti-CD8-PE, anti-CD4-APC and anti-CD3-FITC to measure CTLs in tumor tissue. And cells stained with anti-F4/80-FITC, anti-CD80-APC or anti-CD206-APC and anti-CD11b-PE were used to analyze microphage type I or microphage type II accordingly. The stained cells were tested by flow cytometry (BD C6 Plus). The serum was got by centrifuging blood samples (3000 rpm, 5 min, 4 °C). The IFN-γ cytokine was tested by ELISA kit (Invitrogen), and all options are going according to kit’s introduction.

#### Histology Analysis

Mice were killed, and tumors and organ tissue were harvested immediately. The tissues were fixed by formalin for hematoxylin and eosin staining.

## Results and Discussion

According to the previous study [43], Ag_2_S crystal was formed with bovine serum albumin (BSA), which provided a suitable cavity for Ag_2_S growth under 55 °C. The synthesized Ag_2_S@BSA nanoparticles displayed homogenous sizes with diameter about 5 nm as shown by TEM and DLS analyses (Fig. 1b–d, f). The ratio of Ag_2_S to BSA is about 0.98% (wt%). To conjugate the NO source to the Ag_2_S@BSA NPs, the surface groups of BSA were changed into –SNO group after sulfhydryl reacts with TBN (Fig. 1a). With the -SNO modification, the diameter of the SNO-coupled Ag_2_S@BSA nanoparticles (Ag_2_S@BSA-SNO NPs) exhibited an increase in diameter with an average hydrodynamic size about 50 nm (Fig. 1d), probably due to the self-assemble of Ag_2_S@BSA NPs. Besides, the nanoparticles exhibited good colloidal stability in the PBS (Fig. S1).

**Fig. 2:**
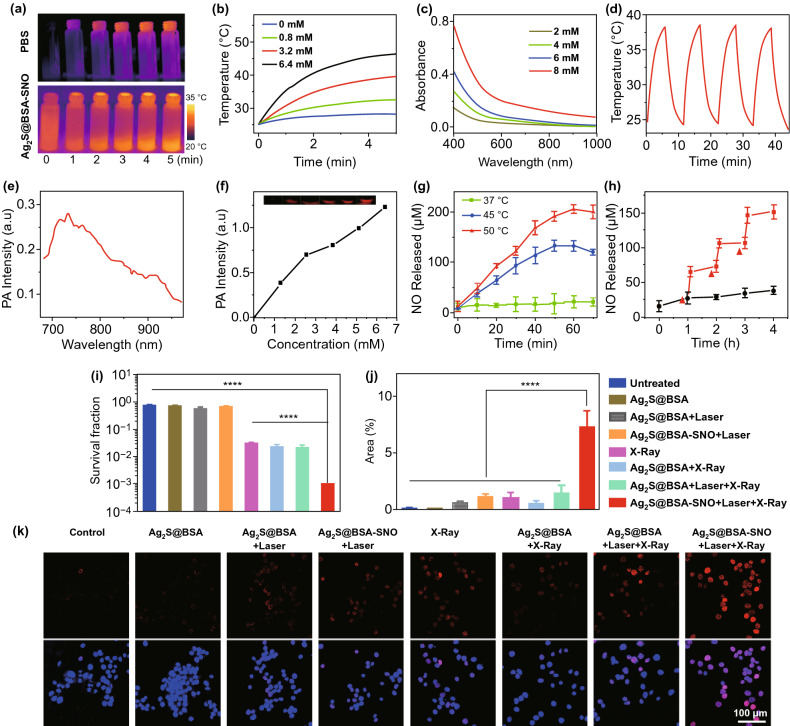
NO-controlled release from Ag_2_S@BSA-SNO nanoparticles and in vitro therapeutic efficiency of X-ray enhanced by Ag_2_S@BSA-SNO nanoparticles. **a** IR images of Ag_2_S@BSA and Ag_2_S@BSA-SNO solution under 808 nm laser irradiation. **b** Temperature change in Ag_2_S@BSA-SNO solution during 808 nm laser irradiation with different concentrations. **c** UV–Vis–NIR spectra of Ag_2_S@BSA-SNO solution with different concentrations. **d** Temperature variations of Ag_2_S@BSA-SNO under irradiation by 808 nm laser (1.5 W cm^−2^). **e** PA intensity of Ag_2_S@BSA-SNO solution at near-infrared region. **f** PA signal of Ag_2_S@BSA-SNO solution (at 730 nm laser). **g** NO released under different temperatures. **h** 808 nm laser irradiation trigger NO release (red triangle means NIR stimulation). **i** Cell cloning statistics. The temperature caused by 808 nm laser is around 42 °C, while the X-ray dose is 6 Gy. **j, k** Statistics and confocal laser scanning microscopy image of DNA damage staining after photothermal and irradiation therapeutics. Red: γ-H2AX signal (dsDNA damage staining); blue: DAPI (nuclear staining). Data are means ± SEM (*n* ≥ 3). *P* value is based on student’s *t* test, with ****P < 0.0001. (Color figure online)

Ag_2_S@BSA-SNO had strong absorption within the visible and NIR regions, making it as a great agent for photothermal therapy and photoacoustic imaging (Fig. 1e). As shown in Fig. 2a, the Ag_2_S@BSA-SNO NPs could be easily heated by NIR laser (808 nm) with the exposure time and concentration dependencies (Fig. 2b). In addition, the solution had a good stability after several times of laser irradiation (Fig. 2d). The Ag_2_S quantum dots also had stable and great photothermal effect under different laser power irradiations according to the previous study [43, 48–51]. The photoacoustic response of Ag_2_S@BSA-SNO under certain wavelength was also examined by VEVO laser imaging system (Fig. 2e). It was shown that 730-nm-wavelength light irradiation resulted in the highest intensity of PA signals. A near-linear relationship was also observed between the concentration of Ag_2_S@BSA-SNO NPs and PA signals intensity (Fig. 2f). As the Ag_2_S@BSA-SNO NPs possessed an NIR-II emission (Fig. S2), which can be used to real-time guide the precision radiotherapy in the future by NIR-II in vivo imaging [43, 48–53]. All these data indicated that Ag_2_S@BSA-SNO could be readily heated by NIR exposure and photoacoustic imaging of tumor.

We next assessed the triggered release of NO from Ag_2_S@BSA-SNO NPs under 808 nm laser irradiation. The generated amounts of NO were determined by a classical Griess assay. As shown in Fig. 2g, we first observed that the NO was generated from Ag_2_S@BSA-SNO NPs when placed under 45 or 50 °C condition, significantly higher than the samples in 37 °C, suggesting that higher temperature could promote the NO release. Importantly, the response release of NO was also achieved when exposed to 808 nm laser (Fig. 2h). Significant amounts of NO were generated from Ag_2_S@BSA-SNO NPs when exposed to 808 nm laser irradiation. In contrast, few NO generation was detected in non-irradiated samples. All the data substantiated that the Ag_2_S@BSA-SNO could be used as NO delivery platform for controlled and precisely release of NO by NIR irradiation.

Next, we investigated the enlargement radiotherapy effects to kill cancer cell by Ag_2_S@BSA-SNO NPs in vitro. In our experiment, the cell viability test by means of cell colony formation assay (CFA) and γ-H2AX-based DNA damage assay were used to evaluate the tumor cells’ proliferation under X-ray irradiation. As control experiment, no appreciable reduction in cell viabilities was noticed for cells incubated with plain Ag_2_S@BSA-SNO NPs by MTT assay at a concentration of 50 µg mL^−1^ (Fig. S3). As shown in Table S1, the nanoparticle solution with suitable concentration had a low hemolysis ratio, which was in the safe range. The hemolysis assay indicated that the nanoparticle did not damage blood cells at the current therapeutic dose. Next, CT-26 tumor cells were treated with Ag_2_S@BSA-SNO NPs (50 µg mL^−1^) 4 h followed by X-ray. The formed cell colonies were counted after 5 days. Various control treatments were also conducted as shown in Fig. 2i. The number of colonies in the control groups treated with Ag_2_S@BSA-SNO NPs + laser did not affect cell proliferation, indicating that neither thermal effect nor generated NO inhibited cell proliferation at the current dose. In addition, the X-ray (6 Gy)-treated group had a slightly lower number of cell colonies, which was consistent with the clinical result. As one kind of heavy metal, Ag_2_S@BSA NPs with X-rays significantly decreased the number of colonies. More importantly, the Ag_2_S@BSA-SNO NPs (+ laser) with X-rays group resulted in the lowest number of colonies. The proliferation of most cancer cells was totally inhibited (the cell survival rate < 2%). To further prove the DNA damage level, the DNA damage staining by γ-H2AX was detected under confocal microscope (Fig. 2j–k and S4). The CT26 cells were immunostained with γ-H2AX at 1 h post-treatments as indicated. Consistently, a marked increase in γ-H2AX foci was observed after Ag_2_S@BSA-SNO NPs (+ laser) with X-rays treatment when compared to control cells. Our results showed that the Ag_2_S@BSA-SNO NPs could maximize radiotherapy effects by Ag_2_S QDs and NO production by NIR exposure.

**Fig. 3:**
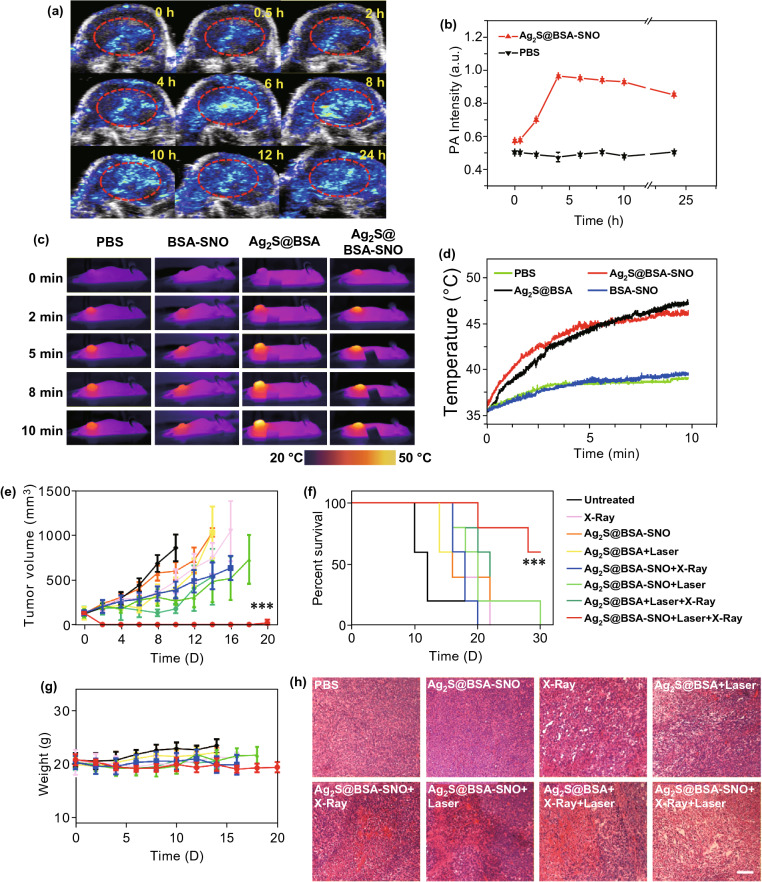
Tumor targeting of Ag_2_S@BSA-SNO nanoparticles and in vivo radio enhancement therapy. **a** Local photoacoustic signal image in tumor at different time points after *i.v.* injection of Ag_2_S@BSA-SNO nanoparticles. **b** Quantitative data of photoacoustic signal according to **a** (PA image of PBS group is shown in supporting information). **c** Corresponding photothermal image of mice injected with different materials under NIR irradiation. **d** Temperature change curve of four groups according to **c**. **e** Tumor growth curves of different groups of mice after various treatments. **f** Survival curves for the treated and control mice. **g** Body weight of mice after different treatments as indicated. **h** H&E sections of tumor tissue after various treatments. Error bar is based on 5–6 mice per group. Data are means ± SEM. Statistical significance was calculated by log-rank (Mantel–Cox) test. ***P < 0.001, and **P < 0.1. Scale bar is 100 µm

We next tested the capacity of Ag_2_S@BSA-SNO nanoparticles accumulated at the tumor site by EPR effect. In vivo PA imaging of tumor was recorded at pre-determined intervals (Fig. 3a). In our experiment, it was observed that in vivo PA signal peaked at 4 h and then gradually fell between 4 and 24 h (Fig. 3a, b) compared with PBS control, indicating that the NPs could accumulate at the tumor by EPR effect. Biodistribution study by ICP of Ag was also consistent with the PA in vivo imaging (Fig. S5). The tumors of mice receiving PBS or Ag_2_S@BSA-SNO nanoparticles administration at 4 h were irradiated by 808 nm laser. Remarkably increased temperature was observed at the tumor site of mice treated with Ag_2_S@BSA-SNO nanoparticles. All the data suggested that Ag_2_S@BSA-SNO nanoparticles accumulated at the tumor site could response to NIR exposure and generate mild heat (~ 45 °C) to trigger NO generation. Notably, we checked the hypoxia of tumor microenvironment post-irradiation (Fig. S6). The immunofluorescence imaging of HIF-1 proved that mild photothermal effect reversed the hypoxia in tumor microenvironment, making tumor more sensitive to RT therapy.

To prove the enhanced anticancer therapy of radiation by our strategy, CT26 tumor-bearing mice received laser irradiation and X-ray irradiation sequentially after being injected with Ag_2_S@BSA-SNO nanoparticles for 4 h. Mice that received other treatments were considered as controls. The tumor size was recorded every two days. As shown in Fig. 3e, compared with untreated group, mice injected with Ag_2_S@BSA-SNO with or without laser exposure showed no obvious tumor growth inhibition efficiency. The X-ray irradiation only slowed the tumor growth. The participation of Ag_2_S, mild heating and released NO further slowed down tumor growth partly. In contrast, the combination therapy (Ag_2_S@BSA-SNO with laser + X ray) significantly inhibited the tumor growth. 60% of mice survived at least 30 days after combination treatment. In contrast, none of the mice survived in any of the control groups after one month (Fig. 3f). Toxic effects of Ag_2_S@BSA-SNO nanoparticles were then evaluated after RT therapy. Body weights of mice were not significantly affected after various treatments (Fig. 3g). Histology analysis of organs also indicated no noticeable organ damage by analysis of H&E staining (Fig. S7), indicating the minimal side effects induced by our strategy. It had been reported that the Ag_2_S quantum dots can be excreted by urine and feces over 60 days without significant toxicity [54–56]. Nevertheless, long-term toxic effect study is needed in the further research.

**Fig. 4:**
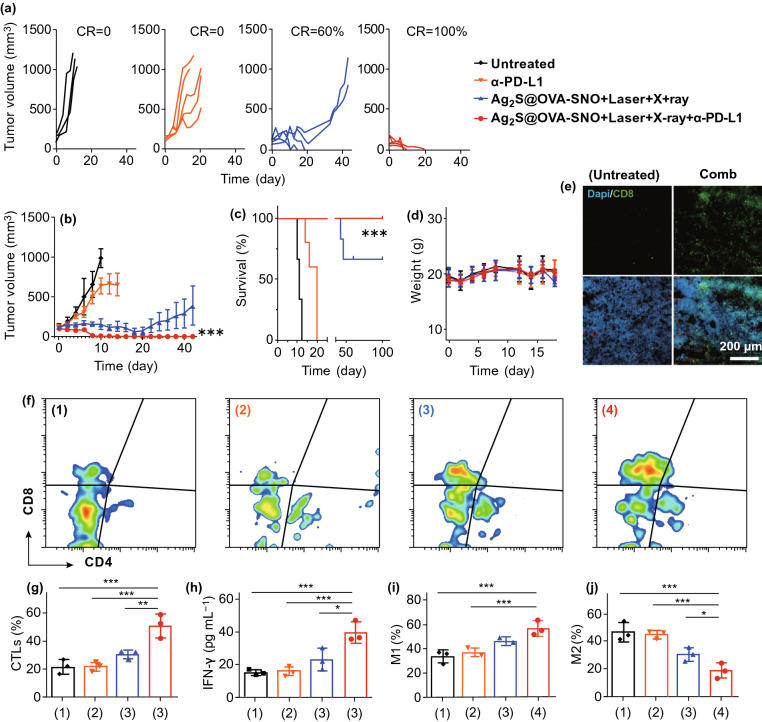
Enhanced radioimmunotherapy in B16-OVA tumor models. **a** Single tumor growth curves of each group after various treatments as indicated. **b** Average tumor growth curves. **c** Survival curves for the treated and control mice. **d** Mice body weight change curves during 20 days after treatment as indicated. **e** Confocal image of tumor tissue sections after 4 days of cancer therapy. Green: CD8 positive cell signal; blue: DAPI (nuclear staining). **f, g** CTLs (CD3+ CD8+) in tumor tissues after 4 days of various treatments by flow cytometric analysis. **h** Cell cytokine levels of serum in different groups mice. **i, j** Flow cytometric analysis of the percentage of macrophages in tumor tissue of different groups. Error bars are based on three mice per group. Data are means ± SEM. For (**g–j**), statistical significance was calculated by one-way ANOVA with Tukey’s post hoc test. For **c**, statistical significance was calculated by log-rank (Mantel–Cox) test. *P < 0.05; **P < 0.01. ***P < 0.005. (Color figure online)

There has been reported that RT, mild heating as well as nitric oxide make tumor more responsive to immunotherapy [57, 58, 11].We next studied the anticancer immune response induced by our enhanced RT therapy. OVA is a T-cell-dependent antigen commonly used as a model protein for studying antigen-specific immune responses in mice. Here, we demonstrated that in addition to BSA, tumor antigen such as OVA can be used to modify our NPs to induce a tumor antigen-specific immune response. We used OVA instead of BSA to modify the Ag_2_S NPs to form the Ag_2_S@OVA-SNO nanoparticles (Fig. S2). B16F10-OVA tumor-bearing B6 mice were treated with anti-PD-L1, Ag_2_S@OVA-SNO + laser with X-ray, or the combination of radioimmunotherapy. Excitingly, we found that anti-PD-L1 further promoted anticancer effects in B16-bearing mice with Ag_2_S@OVA-SNO plus laser and X-ray treatment (Fig. 4a, b). More importantly, 100% of mice were survived at least 100 days (Fig. 4c) without obvious side effects (Fig. 4d), suggesting a high efficacy to treat tumor by our enhanced radioimmunotherapy. Furthermore, tumors were harvested and analyzed by immunofluorescence and flow cytometry on day 4 after treatments. Remarkably, infiltration of CD8+ T cells was observed under fluorescence microscope in the tumor tissue of mice that received combination therapy compared to the untreated mice (Fig. 4e). The analysis by flow cytometry showed that the percentage of CTL cells increased more than twofold compared with controls (Fig. 4f–g). In addition, the Interferon gamma (IFN-γ) level was also found increased after combination therapy (Fig. 4h). M1 and M2-polarized macrophage were also examined. Interestingly, the surface expression of CD80 was up-regulated while the CD206 was down-regulated in comb treated group compared with other control groups, indicating that our strategy could also improve the anticancer effects by regulating the macrophage polarization to M1-like (Fig. 4i, j). Together, these observations suggested that Ag_2_S@OVA-SNO + laser with X-ray in combination with anti-PD-L1 triggered a robust antitumor immune response.

## Conclusions

As a conclusion, we successfully synthesized a NO delivery system based on Ag_2_S QDs for controlled and precisely release of NO to enhance cancer radioimmunotherapy. In this system, protein was used to provide space for Ag_2_S QDs formation, which was coupled with NO donor TBN to generate nitric oxide by NIR exposure. Enhanced radiotherapy caused by nitric oxide and Ag_2_S QDs inhibited the CT26 tumor remarkably in vitro and in vivo. Furthermore, we demonstrated that by tumor antigen OVA modification, the Ag_2_S@OVA-SNO was a promise platform to enhance the antitumor immune response with a high efficacy. 100% survival rate was achieved by our radio-immune combined therapy strategy in mice model. Therefore, such a protein carrier NIR-triggered NO delivery nanoparticles would allow low-dose radiation to treat cancer, enhancing the immunogenic tumor phenotype and promoting the response of immune checkpoint blockade therapy. In addition, the system can not only be applied in cancer treatment but also useful to treat some other diseases by NO delivery.

## Electronic supplementary material

Below is the link to the electronic supplementary material.

Supplementary file1 (PDF 694 kb)
