# Supplementary material for: Near-Infrared Light-Responsive Nitric Oxide Delivery Platform for Enhanced Radioimmunotherapy
Source: Nanomicro Lett. 2020 Apr 24;12:100. doi: 10.1007/s40820-020-00431-3 (PMC7770715; doi:10.1007/s40820-020-00431-3)
Supplement: Supplementary file 1 — Supplementary file1 (PDF 694 kb) [file 40820_2020_431_MOESM1_ESM.pdf]

Supporting Information for

Near-Infrared Light-Responsive Nitric Oxide Delivery Platform for Enhanced Radioimmunotherapy

Xuanfang Zhou[1], Zhouqi Meng[1,*], Jialin She[1], Yaojia Zhang[1], Xuan Yi[2], Hailin Zhou[2], Jing Zhong[2], Ziliang Dong[1], Xiao Han[1], Muchao Chen[1], Qin Fan[1], Kai Yang[2], Chao Wang[1,*]

[1]Institute of Functional Nano & Soft Materials (FUNSOM), Jiangsu Key Laboratory for Carbon-based Functional Materials and Devices, Soochow University, Suzhou, Jiangsu 215123, People's Republic of China

[2]State Key Laboratory of Radiation Medicine and Protection, School of Radiation Medicine and Protection & School for Radiological and Interdisciplinary Sciences (RAD-X), Collaborative Innovation Center of Radiation Medicine of Jiangsu Higher Education Institutions, Soochow University, Suzhou, Jiangsu 215123, People's Republic of China

*Corresponding authors. E-mail: cwang@suda.edu.cn (Chao Wang), mengzhouqi@gmail.com (Zhouqi Meng)

Supplementary Table and Figures

Table S1 Hemolysis ratio of the nanoparticle's solution compared with negative and positive control (3 samples in each group)

	**Negative control**	**Positive control**	**Ag_2S@BSA-SNO (5 mg mL^{-1})**	**Ag_2S@BSA-SNO (10 mg mL^{-1})**	**Ag_2S@BSA-SNO (20 mg mL^{-1})**
**Absorbance intensity**	0.061± 0.001	1,422± 0.016	0.065±0.002	0.069±0.007	0.081±0.006
**Hemolysis**	-	-	0.293%	0.612%	1.4%

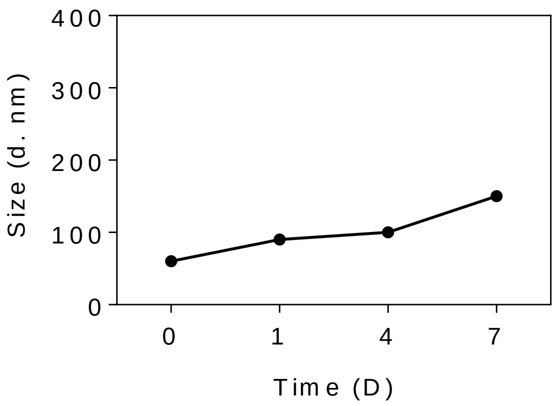

Fig. S1 Colloidal stability of Ag_2S@BSA-SNO nanoparticles in one week

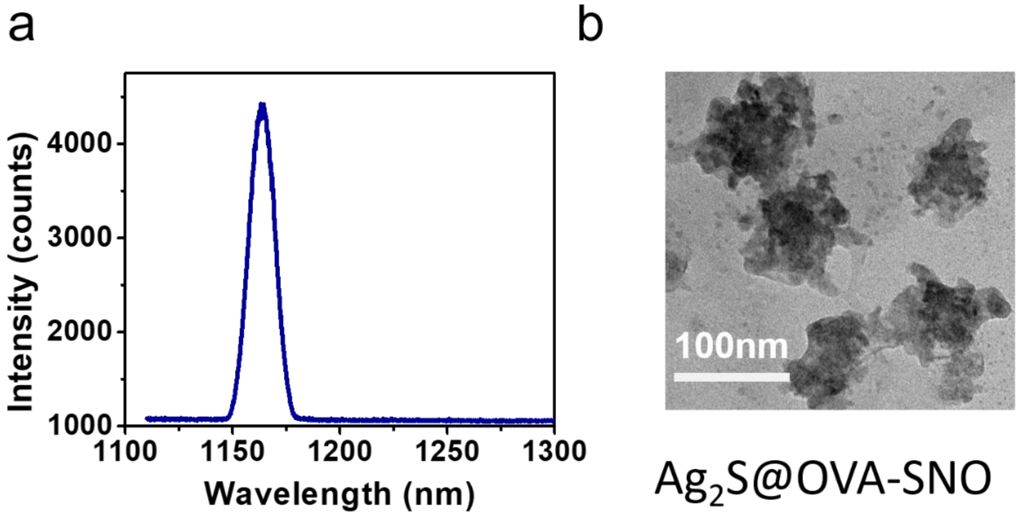

Fig. S2 Characteristics of our nanoparticles. (**a**) Fluorescence of Ag_2S@BSA-SNO solution. (**b**) TEM image of Ag_2S@OVA-SNO nanoparticles

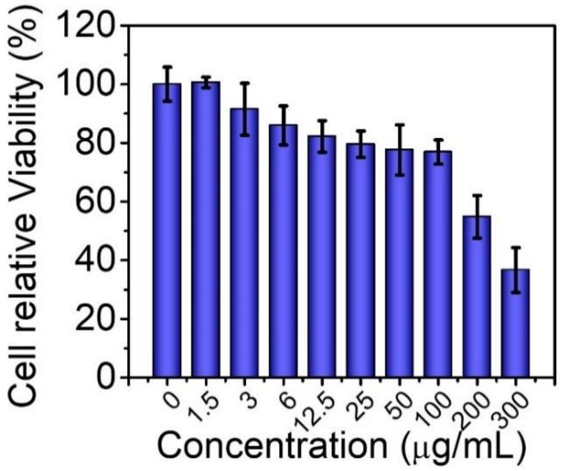

Fig. S3 In *vitro* therapeutic efficiency of our nanoparticles. MTT data of Ag_2S@BSA-SNO nanoparticles after being incubated with materials 24 h

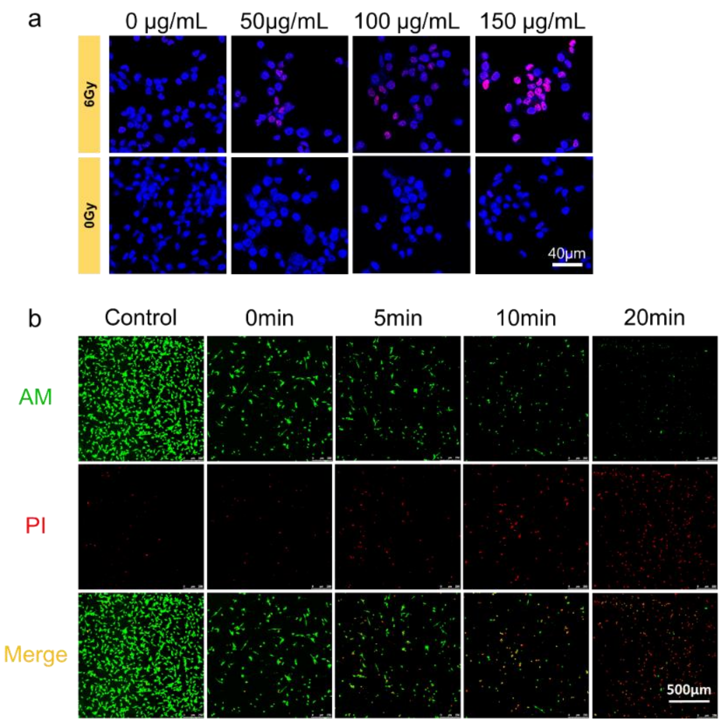

Fig. S4 In *vitro* therapeutic efficiency of our nanoparticles. (**a**) DNA damage staining of cells under different dose and material concentration treatments. Red: γ-H2AX signal (DNA damage); Blue: DAPI (nuclear staining). (**b**) Confocal image of AM/PI double stained experiments. Green: AM staining (live cells); red: PI staining (dead cells)

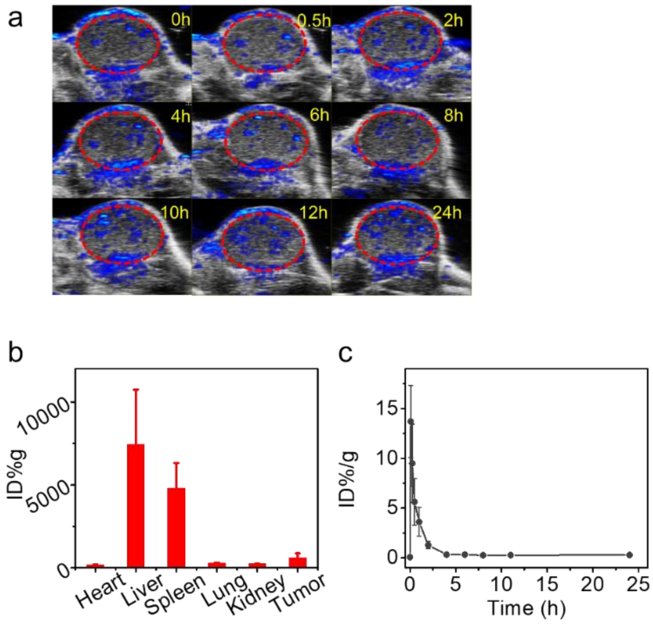

Fig. S5 (**a**) PA image of PBS group. (**b**) Bio-distribution of Ag_2S@BSA-SNO. (**c**) Blood circulation of Ag_2S@BSA-SNO nanoparticles after intravenous injection. The concentrations are determined by element Ag

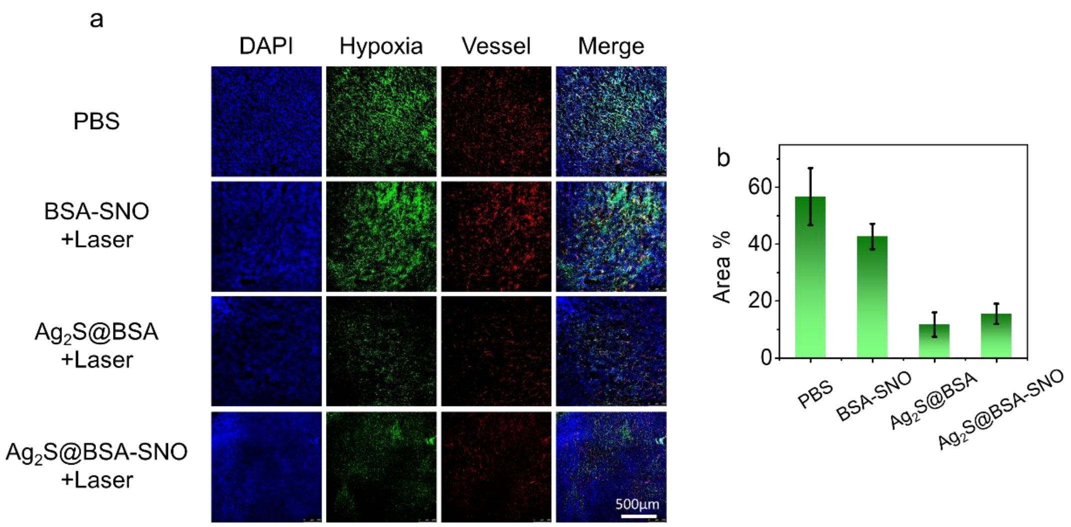

Fig. S6 Hypoxia staining of tumor tissue after various treatments. (**a**) Confocal images of tumor sections Green: hypoxia probe; red: anti-CD31 antibody (blood vessels); blue: DAPI (nuclear staining). (**b**) Statistic data of hypoxia area in the confocal image

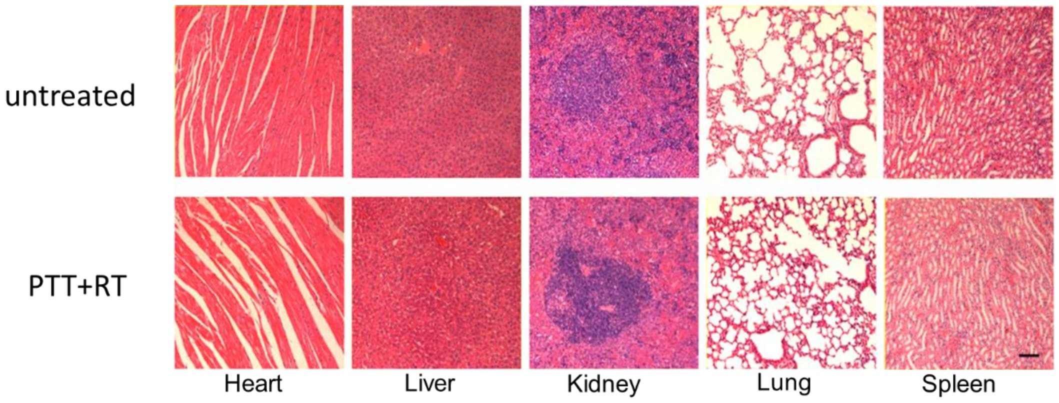

Fig. S7 H&E staining of various tissue sections from different mice
